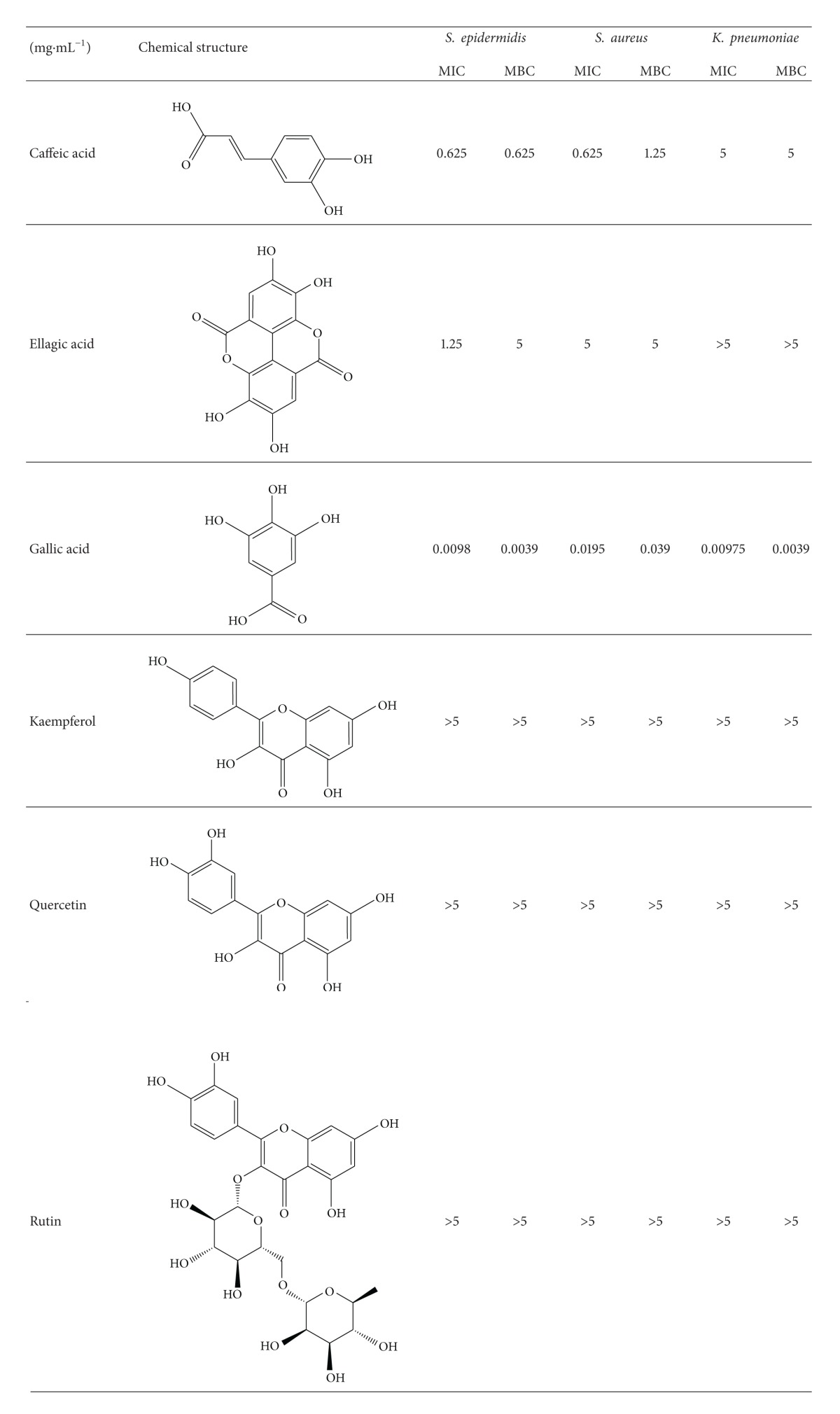# Antibacterial Potential of Northeastern Portugal Wild Plant Extracts and Respective Phenolic Compounds

**DOI:** 10.1155/2014/814590

**Published:** 2014-04-08

**Authors:** Eva Pinho, Isabel C. F. R. Ferreira, Lillian Barros, Ana Maria Carvalho, Graça Soares, Mariana Henriques

**Affiliations:** ^1^CEB, Centre for Biological Engineering, University of Minho, Campus Gualtar, 4710-057 Braga, Portugal; ^2^Centre for Textile Science and Technology (2C2T), University of Minho, Campus Azurém, 4800-058 Guimarães, Portugal; ^3^CIMO/School of Agriculture, Polytechnic Institute of Bragança, Campus de Santa Apolónia, 172, 5301-855 Bragança, Portugal

## Abstract

The present work aims to assess the antibacterial potential of phenolic extracts, recovered from plants obtained on the North East of Portugal, and of their phenolic compounds (ellagic, caffeic, and gallic acids, quercetin, kaempferol, and rutin), against bacteria commonly found on skin infections. The disk diffusion and the susceptibility assays were used to identify the most active extracts and phenolic compounds. The effect of selected phenolic compounds on animal cells was assessed by determination of cellular metabolic activity. Gallic acid had a higher activity, against gram-positive (*S. epidermidis* and *S. aureus*) and gram-negative bacteria (*K. pneumoniae*) at lower concentrations, than the other compounds. The caffeic acid, also, showed good antibacterial activity against the 3 bacteria used. The gallic acid was effective against the 3 bacteria without causing harm to the animal cells. Gallic and caffeic acid showed a promising applicability as antibacterial agents for the treatment of infected wounds.

## 1. Introduction

Skin, the largest human organ, works as a mechanical barrier against environment hazards and is, also, responsible for self-healing, immune surveillance, sensor detection, thermoregulation, and fluid homeostasis [1, 2]. Injuries, caused by extreme temperature, trauma, chronic ulcerations, pressure, or venous stasis, promote disruption of skin integrity allowing the deposition and colonisation of the injury tissue by a wide range of bacteria [3]. Skin and soft tissues infections are typically associated with staphylococci or streptococci, but virtually any microorganism may induce tissue inflammation and immune response [4, 5]. The severity of these infections may range from self-limit superficial infections to life-threatening diseases. The most common treatment is the use of broad-spectrum antibiotics. However, the indiscriminate use of this kind of drugs affects the normal skin flora and may result in multiresistant strains [6]. In order to overcome this issue it is critical to identify new antimicrobial agents.

Plants are a viable, unlimited source of bioactive molecules, including antimicrobial agents which protect them from microorganism, insects, and predators [7–11]. Phenolic compounds belong to these bioactive molecules' group; their pharmaceutical properties and benefits for human health have been demonstrated in several published studies [7, 8]. Anti-inflammatory, antioxidant, and antimicrobial are some of the properties attributed to those molecules [7, 8].

The northeastern region of Portugal, Trás-os-Montes, gathers a wide range of wild plants used on folk pharmacopeia and traditional cuisine. Several ethnobotanical surveys conducted in this region by Mountain Research Centre (CIMO)-ESA, Polytechnic Institute of Bragança, selected some of these wild plants as potential source of natural antimicrobial agents. 

The present work aims to select phenolic compounds identified on extracts of selected wild plants from northeastern region of Portugal to be applied as antibacterial agents on the treatment of infected wounds. The antibacterial activity of 8 phenolic extracts and 6 phenolic compounds was tested against* Staphylococcus epidermidis*,* Staphylococcus aureus*, and* Klebsiella pneumoniae*, usually associated with skin and soft tissue infections. The influence of the most effective phenolic compounds on human fibroblasts was, also, evaluated.

## 2. Material and Methods

### 2.1. Phenolic Extracts and Compounds

The plant samples and their phenolic extracts were obtained as described by Barros:* Asparagus acutifolius* (shoots), Aa, and* Bryonia dioica* (young stems), Bd [12];* Cytisus multiflorus* (flowers), Cm, and* Sambucus nigra* (flowers), Sn, [13];* Rosa micrantha* (flowers), Rm,* Filipendula ulmaria* (inflorescences), Fu, and* Castanea sativa* (upright catkins during anthesis), Cs [14]; and* Cistus ladanifer *(leaves), Cl [15]. The phenolic characterization of the extracts is also described on the publications mentioned above. Six different phenolic compounds, recovered from those plants, were pointed out as the main ones: 3 phenolic acids (caffeic, ellagic and gallic acids) and 3 flavonoids (kaempferol, quercetin and rutin) [12–15].

### 2.2. Strains and Growth Conditions

The antibacterial activity of the phenolic extracts and compounds was tested against 3 bacteria:* Staphylococcus epidermidis *(ATCC 12228),* Staphylococcus aureus* (ATCC 6538), and* Klebsiella pneumoniae* (ATCC 11296). The bacteria were grown in tryptic soy agar (TSA, Merck, Germany) for 24 h at 37°C. The cells were inoculated in tryptic soy broth (TSB, Merck, Germany) and incubated for 18 h at 37°C under agitation (120 rpm). Subsequently, bacterial concentration of each strain was adjusted to 1 × 10^6^ cells·mL^−1^, via absorbance readings and the corresponding calibration curve.

### 2.3. Disk Diffusion Assay

The antibacterial activity of the extracts or compounds against the 3 bacteria was assessed, first, by the disc diffusion method described by the National Committee for Clinical Laboratory Standards (NCCLS), M2-A8 document [16], with some modifications. The TSA was the nutritive media used and it was prepared according to the instructions of the manufacturer. Afterwards, 200 *μ*L of each inoculum (1 × 10^6^ cells·mL^−1^) was spread on the solid media plates (90 mm Petri dishes). Sterile filter paper disks (“Blank Discs,” Liofilchem, Roseto, Italy, 6 mm in diameter) were placed over the petri dish and impregnated with 20 *μ*L of each extract (200 mg·mL^−1^) or compounds (5 mg·mL^−1^). The plates were incubated at 37°C for 24 h. Thereafter, the size of the halo from the inhibition growth was measured.

### 2.4. Susceptibility Assay: Minimal Inhibitory Concentration (MIC) and Minimal Bactericidal Concentration (MBC)

MIC and MBC were obtained according to the method described by Wiegand et al. [17], an adaptation of the standard method published by Clinical and Laboratory Standards Institute (CLSI) and the European Committee on Antimicrobial Susceptibility Testing (EUCAST) [18], using the broth microdilution procedure. Thus, a work solution of 20 mg·mL^−1^ of each extract and 10 mg·mL^−1^ of each compound were prepared in sterile distilled water. The 96-well plate (Orange Scientific, Braine-l' Alleud, Belgium) was prepared by adding 100 *μ*L of a solution of each extract/compound to a final concentration of 20 mg·mL^−1^/10 mg·mL^−1^ to the first well. Then serial dilutions (1 : 10) were made with MHB (Mueller-Hinton broth, Merck, Germany) in the other wells, to final volume of 50 *μ*L. At each well, 50 *μ*L of each bacterium was added (*S. epidermidis, S. aureus, *and* K. pneumoniae*). The extracts concentration tested ranged between 0.02 and 10 mg·mL^−1^ and the phenolic compounds from 0.01 to 5 mg·mL^−1^. Drug-free and bacteria controls were also included. The plates were incubated for 24 h at 37°C.

The MIC value was determined by the observation of the concentration that did not show any growth, by contrast with the bacteria control. The MBC, number of viable cells, was assessed by determination of the number of colony forming units (CFUs). The CFUs were measured by plating 10 *μ*L of cell suspension from each well onto TSA and incubated for 24 h at 37°C.

The procedure was made in triplicate for each extract, compound and bacteria combination, in, at least, 3 independent assays.

### 2.5. Cytotoxicity Determination

Fibroblast 3T3 (CCL 163) from American Type Culture Collection was used in this study. Cells were cultured in Dulbecco's modified Eagle's medium (DMEM) supplemented with 10% of foetal bovine serum and 1% penicillin/streptomycin at 37°C, 5% CO_2_. After achieving the confluence, cells were passed at the density of 1 × 10^5^ cells·mL^−1^, using trypsin.

To assess the effect of the compounds on the cellular viability, the cells were seeded at the density of 5 × 10^5^ cells·mL^−1^ (24 well plate) in 1 mL of DMEM complete medium. After 24 h, the medium was replaced by 500 *μ*L of fresh one and 500 *μ*L of compounds, at twofold of the desired concentrations dissolved in PBS. The plates were incubated for 24 h at 37°C and 5% CO_2_. Afterwards, the medium was removed and a mixture of 20 *μ*L of MTS [3-(4,5-carboxymethoxyphenyl)-2-(4-sulfophenyl)-2H-tetrazolium] (Promega) and 980 *μ*L of DMEM without phenol was added to each well. After 1 h, the absorbance value was measured at 490 nm and the results were expressed as percentage of viable cells (%), using the number of cells grown on wells without compounds as controls.

The procedure was made in triplicate for each compound, at least, in 3 independent assays.

## 3. Results and Discussion

The emergence of multiresistant strains of pathogenic and opportunistic bacteria is correlated with the widespread use of broad-spectrum antibiotics for treatment of skin and soft tissue infections. Therefore, the search for new drugs and new sources of antibacterial agents is of outmost importance [19]. Natural sources, such as plants, have been explored and gained prominence, since they offer many advantages when compared to the synthetic ones. For instance, they show high levels of biocompatibility and availability and low toxicity [7–10]. Currently, polyphenolics are the major group of interest in view of their anti-inflammatory, antimicrobial, antiviral, and antioxidant properties [20].

The antibacterial activity of phenolic extracts, from medicinal Portuguese plants, and, also, of polyphenolic compounds, identified on those extracts, was assessed by both qualitative and quantitative methods. The disk diffusion assay is a qualitative method, which allows a first screening of the potential antibacterial agents. However, this method presents some issues regarding the capacity of the active molecules to diffuse into the agar, and so, a quantitative method such as the MIC and MBC determination should be used in order to obtain more accurate results. The MIC is defined as the lowest concentration of the antibacterial agent that inhibits the visible bacteria growth observed with unaided eye, and the MBC is the minimal concentration of the antibacterial agent required to destroy most of the viable bacteria (reduction of 3 logs of growth) for a given set of conditions [21]. It is important to refer to the fact that the extracts and compounds present some colouration, which may lead to some misleading of the MIC values. Therefore, the MBC determination is crucial for a complementary analysis of the antibacterial properties of the phenolic extract/compounds [19].

### 3.1. Antimicrobial Activity of the Extracts

**Figure 1:**
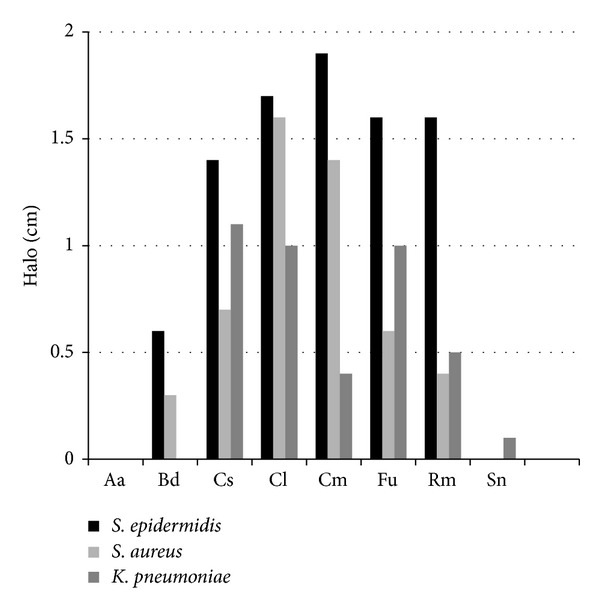
Values of the halo dimension (disk diffusion assay) for each extract (200 mg·mL^−1^) for the 3 bacteria. The halo size was calculated by deducting the size of the disk (0.6 cm). Aa:* A. acutifolius*; Bd:* B. dioica;* CS:* C. sativa; *Cl:* C. ladanife; *Cm:* C. multiflorus;* Fu:* F. ulmaria*; Rm:* R. Micrantha; *and Sn:* S. nigra.*

A preliminary assay using phenolic extracts of Portuguese medicinal plants was made, in order to identify phenolic compounds from those extracts. The chemical characterization of the phenolic extracts was described in previous works [12–15]. Both qualitative and quantitative analyses were made to the antibacterial activity of 8 phenolic extracts. From those extracts, only 5 (Cs, Cl, Cm, Fu, and Rm) were capable of reducing the growth of the 3 bacteria used (Figure 1). These extracts were selected as the most promising ones and their MIC and MBC values were assessed.

Thus, these extracts were selected as the most promising ones. As the disk diffusion assay is based on the measurement of the growth inhibition halo, which is dependent on the antibacterial agent ability to diffuse trough agar, quantitative complementary assays were also performed.

The MIC and MBC values revealed that the Cs and Cl had a similar effect on the bacteria, being more effective against* K. pneumoniae* and* S. epidermidis *(MIC and MBC 0.625 mg·mL^−1^ (for both species)) and less effective against* S. aureus *(MIC 1.25 and MBC 2.5 mg·mL^−1^). The Fu and Rm extracts, also, showed similar effect against the bacteria; namely, 2.5 mg·mL^−1^ was capable of reducing completely the 3 bacteria growth when exposed to these 2 extracts. Since Cm phenolic extract had MIC and MBC values higher than 10 mg·mL^−1^, this extract was not used for further analysis.

### 3.2. Antimicrobial Activity of the Phenolic Compounds

Six phenolic compounds identified in the extracts of Cl (ellagic acid, kaempferol, and gallic acid), Fu (caffeic acid, kaempferol, rutin, and gallic acid), Cs (gallic acid and rutin), and Rm (kaempferol) were selected for further analysis. Those compounds are all polyphenolics and can be placed into two groups: (1) phenolic acids and (2) flavonoids. Caffeic, gallic, and ellagic acid belong to the first group and the remaining compounds (kaempferol, quercetin, and rutin) fit into the flavonoids group.

**Figure 2:**
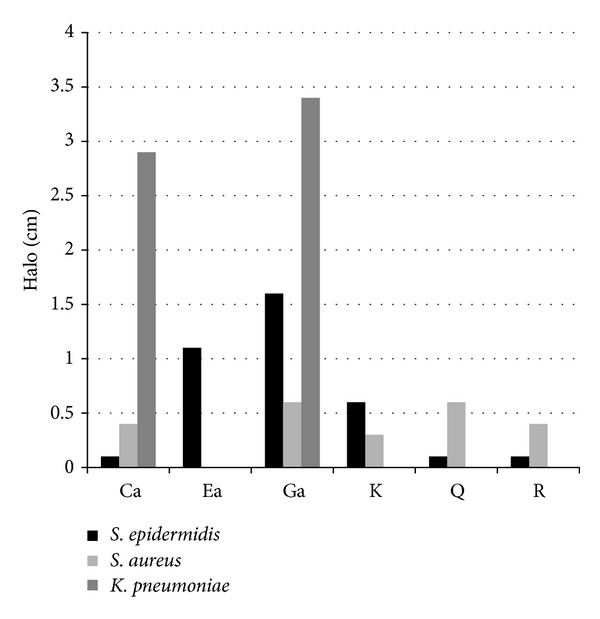
Values of the halo dimension (disk diffusion assay) for each extract (5 mg·mL^−1^) for the 3 bacteria. The halo size was calculated by deducting the size of the disk (0.6 cm). Ca: caffeic acid; Ea: ellagic acid; Ga: gallic acid; K: kaempferol; Q: quercetin; and R: rutin.

The disk diffusion assay of the phenolic compounds (Figure 2) demonstrated that they can inhibit the growth of* S. epidermidis*, with the gallic acid being the most efficient and caffeic acid, rutin, and quercetin being the least efficient. The compounds present a similar halo size against* S. aureus* with the exception of ellagic acid that did not change the bacteria growth. Gallic acid and caffeic acid were the only phenolic compounds tested capable of inhibiting the Gram-negative bacteria (*K. pneumoniae*). Due to the different and interesting results of the compounds in the diffusion assay, it was decided to determine the MIC and MBC of all of them (Table 1).

Flavonoids are a group of polyphenolic molecules from plant source with many biological properties already studied [22, 23]. The flavonoids antibacterial capacity is based on their ability to complex with extracellular and soluble proteins and to destroy the bacteria cell wall by interacting with essential enzymes responsible for maintaining the stability of this structure [7, 19]. However, in the conditions tested, the flavonoids selected (kaempferol, quercetin, and rutin) had no effect on the bacteria growth for concentrations under 5 mg·mL^−1^.

Our results, similar to Penna's [24], suggest that kaempferol has no activity under 5 mg·mL^−1^ against* S. aureus*. Additionally, Fattouch et al. [25] showed that kaempferol presented activity only at a concentration of 10 mg·mL^−1^. Regarding quercetin antimicrobial activity, El-Gammal and Mansour [26] described that its MIC for* S. aureus* was 37 *μ*g·mL^−1^. Fattouch et al. [25] achieved a MIC and MBC for quercetin of 10 mg·mL^−1^ for the same bacteria, which corroborates our results. In this case, the differences among results can be justified by the methods used. For instance, Fattouch et al. [25] used the microdilution method, the same procedure used in this work; however, El-Gammal and Mansour [26] used a method dependent on the diffusion capacity of the compounds which justify the differences on the MIC of the quercetin. In the case of rutin, some authors described that 0.5 or 4 mg·mL^−1^ [27, 28] was enough to destroy all cells of* S. aureus,* but our results suggest that rutin is not capable of reducing the total number of viable cells of* S. aureus* for concentrations under 5 mg·mL^−1^. Bisignano et al. [27] obtained the MIC of rutin by the macrodilution method and, also, the phenolic compound was dissolved in DMSO; Orhan et al. [28] used the microdilution test, but they dissolved the rutin in ethanol-hexanol. In both situations, the MIC values described may be due to the solvents used for rutin dissolution and, also, the MBC should be assessed as a complementary method.

Concerning the phenolic acids tested, ellagic acid was not able to inhibit the growth of the 3 bacteria used, although its MIC for* S. epidermidis *was 1.25 mg·mL^−1^. The ellagic acid has been described as antibacterial agent and its mechanism of action is related to the capacity to interact with enzymes, inhibiting their action and interactions with proteins [29]. Ohemeng et al. [30] showed that the MIC of the ellagic acid against the bacteria* S. epidermidis* and* S. aureus* was 0.125 mg·mL^−1^ and Thiem and Goślińska [31], besides MIC, also determined the MCB of this phenolic acid against the same bacteria and reported the values of 0.63 and 2.5 mg·mL^−1^, respectively.

In the literature, the gallic acid and the caffeic acid showed the same antibacterial mechanism, related to their similar structure (Table 1). These phenolic acids disrupted the bacteria cell by hyperacidification of the plasma membrane via proton donation and acidification of the intracellular cytosolic; this low pH can inhibit the enzyme H^+^-ATPase necessary for the ATP production [32–34]. Our results demonstrated that the gallic acid and the caffeic acid had effect against the 3 bacteria tested. However, the first phenolic was active against both Gram-positive and Gram-negative bacteria with concentration on the range of *μ*g·mL^−1^ and the caffeic acid activity was only detected when concentrations between 0.625 and 5 mg·mL^−1^ were used. Moreover, the* K. pneumoniae* was the most resilient bacteria to the caffeic acid (Table 1).

Most of the published works regarding the antibacterial activity of gallic and caffeic acids use the disk diffusion assay method without any quantitative analysis for complementary analysis [32, 35–37]. This which may lead to misleading results related to the capacity of these molecules to diffuse into the solid medium. Regarding the caffeic acid activity, Kwon et al. reported formation of halo when in contact with Gram-positive and Gram-negative bacteria for 50 mg·mL^−1^ [32, 37]. Gutiérrez-Larraínzar et al. [35] obtained a MIC value for* S. aureus* of 561 *μ*g·mL^−1^ of gallic acid and Binutu and Cordell [38] had a MIC of 250 *μ*g·mL^−1^, 10 and 7 times higher, respectively, than the value attained in this work despite of the fact that method used was the same. The differences of the MIC value may rely on lower temperature (35°C instead of 37°C) used for the growth of* S. aureus *by Gutiérrez-Larraínzar et al. [35] or in a misleading caused by the colouration of the gallic acid [19]. 

The differences found on the results published may be due to the diverse methods applied in each work to assess the MIC and MBC and/or interferences on the MIC and MBC procedure, such as variations on the volume and concentration inoculum, source of the flavonoid (natural or commercial), salts formation, and precipitation leading to misleading results [19].

### 3.3. Effect of Gallic and Caffeic Acid on 3T3 Fibroblast Growth and Adhesion

Besides the antibacterial activity of natural molecules, the knowledge of their effect on the human cells is also crucial. Therefore, to predict the effects of the most promising phenolic compounds (gallic and caffeic acid) on animal cells, a preliminary assay of cytotoxicity was made.

**Figure 3:**
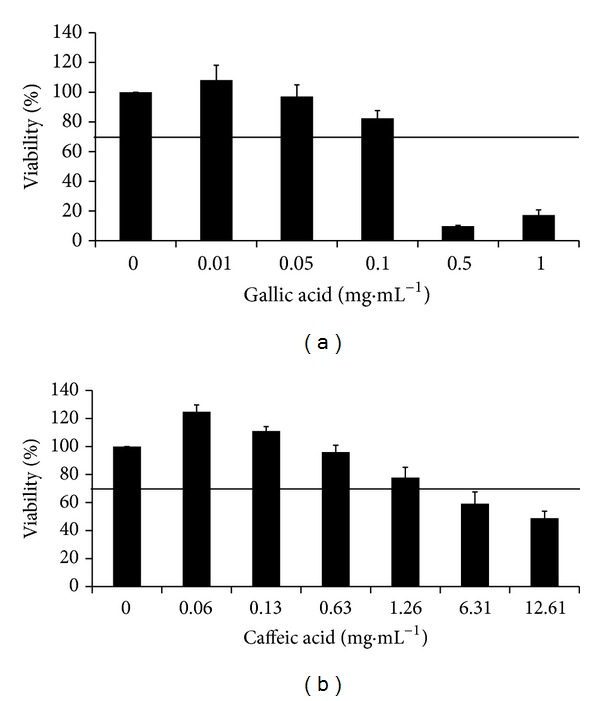
The viability of cells after 24 h of contact with gallic acid (a) and caffeic acid (b) dissolved in PBS, measured with an MTS assay. All data is expressed as mean + standard deviation (*n* = 9). The line indicates 70% of cell viability; when higher values were obtained the compound was considered nontoxic to the cells.

The gallic acid showed no toxicity to the fibroblast when concentrations between 0.01 and 0.1 mg·mL^−1^ were used (Figure 3(a)). In fact, the lowest concentration induced an increase on the number of viable cells measured. However, for concentrations above 0.1 mg·mL^−1^, gallic acid became toxic.

The effect of caffeic acid on the viability of cells was dose dependent. The caffeic had no significant influence on the cells growth for concentrations between 0.06 and 1.26 mg·mL^−1^, but a reduction greater than 30% of viability was measured when 6.31 mg·mL^−1^, or higher, was applied.

Both phenolic compounds have been described as potent antioxidant, and as consequence they exert some chemopreventive effects on animal cells [39, 40]. However, their antioxidant activity is based on oxidation-reduction reactions that are reversible and dependent on concentration. Therefore, these phenolics can act both as antioxidant and prooxidant, depending on the reaction conditions [39, 41]. This explains the fact that for higher concentrations gallic and caffeic acids induce major reduction on the cells viability. Additionally, gallic acid was more toxic, since less than 20% of cells were capable of surviving for the concentrations between 0.5 and 1 mg·mL^−1^ (Figure 3(a)). For the same range of the concentrations, the cells tolerated the caffeic acid (Figure 3(b)). Our findings corroborate the fact that the predisposition to act as prooxidant is directly proportional to the number of hydroxyl groups in the molecule; gallic acid has 4 hydroxyl groups and caffeic only 3 (Table 1).

Nerveless, the gallic acid can be used as antibacterial agent against the bacteria tested without causing any damage to the animal cells, since its MBC was 0.04 mg·mL^−1^ and at this range of concentrations the percentage of viable cells measured was higher than 70%; above this limit the compounds are safe for humans based on the ISO 10993-5:2006. In the case of caffeic acid, the concentration capable of destroying all the bacteria was, also, toxic to the fibroblast, which may suggest that the mechanism involved on the antibacterial action is the same as one that causes damage to animal cells.

## 4. Conclusion

The results presented in this work highlight the potential of phenolic extracts from wild northeast Portuguese plants as antibacterial agents, as well as some of their phenolic compounds.

Overall, extracts from* Cistus ladanifer*,* Cytisus multiflorus*,* Castanea sativa*,* Filipendula ulmaria,* and* Rosa micrantha* were capable of inhibiting the growth of the 3 bacteria commonly isolated from skin and soft tissue infections. Moreover,* Cistus ladanifer*,* Castanea sativa*,* Filipendula ulmaria,* and* Rosa micrantha* revealed promising antibacterial effects against* K. pneumoniae*,* S. epidermidis,* and* S. aureus*, in concentrations between 0.625 and 2.5 mg·mL^−1^.

From those extracts, 6 phenolic compounds were selected. The flavonoids (kaempferol, quercetin, and rutin) were capable of inducing halo formation on Gram-positive bacteria. However, the quantitative assay of the flavonoids demonstrated that they were not active for concentrations below 5 mg·mL^−1^. Regarding the phenolic acids, the ellagic acid was only active against* S. epidermidis*, but gallic and caffeic acids showed good antibacterial activity against the 3 bacteria at low concentrations. Thus, their effect on fibroblast proliferation was assessed and revealed that caffeic acid has dose-response cytotoxicity and can be considered safe for concentrations less than 6.31 mg·mL^−1^. Regarding gallic acid, at the lowest concentration it promoted the proliferation of fibroblast but for concentrations above 0.1 mg·mL^−1^ it became toxic. Nevertheless, conjugating the antibacterial and cytotoxicity results, it could be pointed out that gallic acid can be used safely presenting antibacterial activity against the 3 bacteria.

Therefore, the present work pretends to be a starting point to the use of phenolic compounds from northeastern Portugal plants on the treatment of infected wounds, instead of large-spectrum antibiotics.

## Figures and Tables

**Table 1:** MIC and MBC of the selected compounds for each of the bacteria (5 × 10^5^ cells·mL^−1^).